# Measuring disadvantage in the early years in the UK: A systematic scoping review

**DOI:** 10.1016/j.ssmph.2022.101206

**Published:** 2022-08-15

**Authors:** A. Clery, C. Grant, K. Harron, H. Bedford, J. Woodman

**Affiliations:** a Great Ormond Street Institute of Child Health, University College London, London, WC1N 1EH, United Kingdom; b Institute of Epidemiology and Health Care, University College London, London, WC1E 6BT, United Kingdom; c Thomas Coram Research Unit, Social Research Institute, University College London, London, WC1H 0AA, United Kingdom

**Keywords:** Early years, Disadvantage, Scoping review, Child health

## Abstract

**Background:**

The relationship between disadvantage and child health in the early years is well established. For this evidence base to most helpfully inform services, we need to better understand how disadvantage is conceptualised and measured in the literature. We aimed to conceptualise disadvantage measured in child health literature and explore the associations between disadvantage and child health using these measures.

**Method:**

We conducted a scoping review using systematic methods to identify key concepts of disadvantage used in empirical child health literature. We searched MEDLINE, Scopus, and grey literature for studies exploring the association between disadvantage and child health outcomes for children aged 0–5 in the United Kingdom. We extracted and analysed data from 86 studies.

**Results:**

We developed a framework describing two domains, each with two attributes conceptualising disadvantage: level of disadvantage indicator (individual and area) and content of disadvantage indicator (social and economic). Individual-level measures of disadvantage tended to identify stronger associations between disadvantage and child health compared with area-level measures.

**Conclusion:**

The choice of disadvantage indicators, particularly whether individual- or area-level, can affect the inferences made about the relationship between disadvantage and child health. Better access to individual-level disadvantage indicators in administrative data could support development and implementation of interventions aimed at reducing child health inequalities in the early years.

## Highlights

• Measurement of disadvantage in child health is wide-ranging and multi-dimensional.
• Social, economic, individual, and area-level are key concepts of disadvantage.
• Area-level measures underestimate the association between disadvantage and child health.
• Individual-level disadvantage indicators are needed in administrative data.
• Policymakers planning child health interventions should measure disadvantage carefully.

## 1 Introduction

Reducing child health inequalities is a key target for policy and research globally. In the United Kingdom (UK), interest has focussed specifically on the early years (Department of Health and Social Care, 2021). Evidence suggests intervening at this early time not only improves development and outcomes in childhood and adult life, but is also of economic benefit (Department of Health and Social Care, 2021; Early Intervention Foundation, 2018).

Historically, implementing interventions to reduce child health inequalities has not always been successful. This is potentially due to the challenge in the UK of identifying appropriate target populations that can benefit most from interventions (C. Law, Parkin, & Lewis, 2012). This requires effective measurement of disadvantage. However, due to variation and ambiguity in how disadvantage is measured, there is uncertainty in how best to target interventions.

There is a long history of conceptualising, defining, and measuring disadvantage in a range of disciplines and for different outcomes of interest. In the social and political sciences, disadvantage has been conceptualised in the context of inequalities across social groups (class, gender, ethnicity, migration, and disability), and unequal opportunity (Platt, 2011; Wolff & de Shalit, 2007). As identified by Platt (2011), disadvantage can also be conceptualised in relation to experiences throughout the life course, such as income, education, and housing. An extensive literature describes how to measure these in social epidemiology and health research, such as occupation-based measures, composite measures and those specifically pertaining to income and poverty (Betson & Warlick, 2006; Connelly, Gayle, & Lambert, 2016; Galobardes, Lynch, & Smith, 2007). These have also been the focus of measuring disadvantage in the child health literature (Cooper & Stewart, 2021; Graham & Power, 2004; Pearce, Dundas, Whitehead, & Taylor-Robinson, 2019; Wickham, Anwar, Barr, Law, & Taylor-Robinson, 2016).

An additional consideration in measuring disadvantage is the availability of indicators. Given their sensitive nature, they can be limited with proxy indicators are often used in their place. In health research, this often relies on area-based measures using an individual's postcode or other area-level data. Research in North America suggests that area-level measures of socioeconomic status are sub-optimal or even inappropriate proxies for individual-level measures when looking at mortality and child health outcomes (Buajitti, Chiodo, & Rosella, 2020; Moss, Johnson, Yu, Altekruse, & Cronin, 2021; Pardo-Crespo et al., 2013). In the UK, the Index of Multiple Deprivation (IMD) is a readily available, area-level measure of disadvantage, which can be linked to administrative health data and is therefore frequently used in research (Ministry of Housing Communities & Local Government, 2019). Given what previous research has shown, understanding how such area-level measures are used in early years research, policy, and planning in the UK is of considerable interest.

Childhood disadvantage is not a clearly defined concept and there is known variation in how it is measured. Therefore, this review aims to (1) conceptualise how disadvantage is measured in the UK child health literature, and (2) explore the associations between disadvantage and child health and development outcomes relevant to the early years.

## 2 Methods

**Fig. 1:**
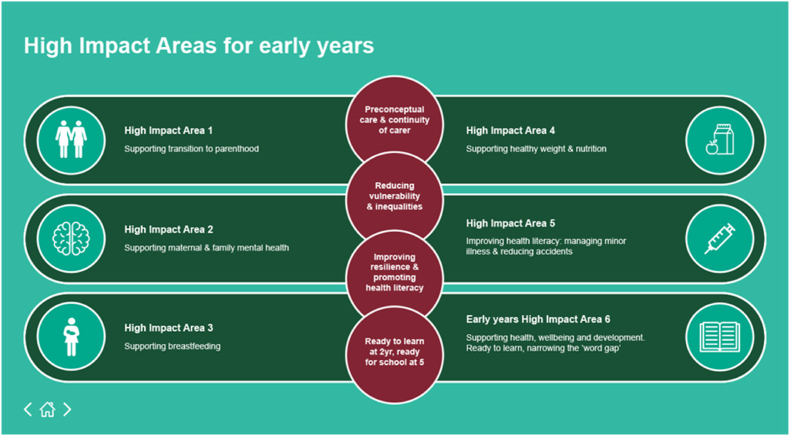
Healthy Child Programme early years high impact areas Image taken from: https://www.gov.uk/government/publications/commissioning-of-public-health-services-for-children/health-visiting-and-school-nursing-service-delivery-model.

We used a systematic scoping review methodology to map, define, and clarify key concepts of disadvantage in the child health literature (Arksey & O'Malley, 2005; Peters et al., 2015). We scoped the literature for studies that explored the association between disadvantage and child health outcomes for children aged 0–5 in the UK. We focused on studies that measured the child health outcomes outlined in England's Healthy Child Programme (Fig. 1). This is the national, universal public health early intervention programme for the early years (Department of Health, 2009). We focus on areas 3–6 (breastfeeding, healthy weight and nutrition, reducing minor illnesses and accidents, and child development). We did not explore areas 1 and 2 (parenthood and maternal mental health) as these outcomes do not focus on child health (Public Health England, 2021).

We used the Preferred Reporting Items for Systematic Reviews and Meta-Analyses: Extension for Scoping Reviews (PRISMA-ScR) guidelines and followed the Arksey and O'Malley framework (Arksey & O'Malley, 2005; Tricco et al., 2018). EndNote and Microsoft Excel were used for reference management.

### 2.1 Eligibility criteria

The following criteria had to be met for a study to be included in the review:

1. Conducted exclusively in the UK and written in English
2. Published since 2009, when the HCP was introduced
3. Outcomes measured primarily in children aged 0–5 years
4. Reported the association between disadvantage and the child health outcome
5. Reported at least one of the child health outcomes of interest

Studies were excluded if they made comparisons between the UK and other countries, measured children as a whole population (0–18 years), or explored other child health outcomes.

### 2.2 Search strategy

We searched MEDLINE (Ovid) and Scopus for peer-reviewed journal articles published between January 1, 2009 and December 7, 2020. We also searched the following sources for grey literature: Social Care Online, former Public Health England, The Nuffield Foundation, The Children's Commissioner, Local Government Association, The Joseph Rowntree Foundation, and National Children's Bureau.

For database searching, we used four key concepts: disadvantage AND children under 5 AND child health outcomes AND studies from the UK. For the grey literature searching, website search engines were searched using broader terms compared to the database searches, to avoid missing key literature. The search terms used depended on the organisation's expertise, e.g., where organisations didn't have a child health focus, search terms concentrated on child health, whereas for organisations which already had a child health focus, we searched key concepts of disadvantage. Full details of the search strategy are provided in the supplementary material.

### 2.3 Data extraction

Once all articles were identified, and duplicates removed, two independent reviewers (AC and CG) screened all abstracts for relevance, through application of the inclusion criteria. We resolved any disagreements through discussion and a final list of full-text records for screening was established. Full-text copies of the relevant articles were obtained and read in full by the primary reviewer (AC), and the second reviewer (CG) screened a random sample of 20% of these full-texts. Those that met all inclusion criteria were included for analysis.

From included studies we extracted: the terms used to describe disadvantage, the indicator(s) used to measure disadvantage, the level at which the indicator was measured (individual or area), and the association between disadvantage and child health outcomes (extracted from results tables based on statistical estimates and the authors’ conclusions).

### 2.4 Data analysis

#### 2.4.1 Concept analysis

We developed a concept analysis as follows: (1) identified individual concepts used to measure disadvantage, (2) identified the main attributes that were used to define each concept, (3) organised the concepts according to their attributes, grouping similar concepts, identifying relationships between them, and mapping them into a conceptual framework. Any concepts that were only used in a single study were excluded from the framework.

#### 2.4.2 Association analysis

We looked in greater detail at a subgroup of studies that measured both individual-level and area-level indicators of disadvantage. In these, we explored their evidence for associations between disadvantage and child health outcomes, comparing the results between the two types of indicators of disadvantage.

## 3 Results

### 3.1 Search results

**Fig. 2:**
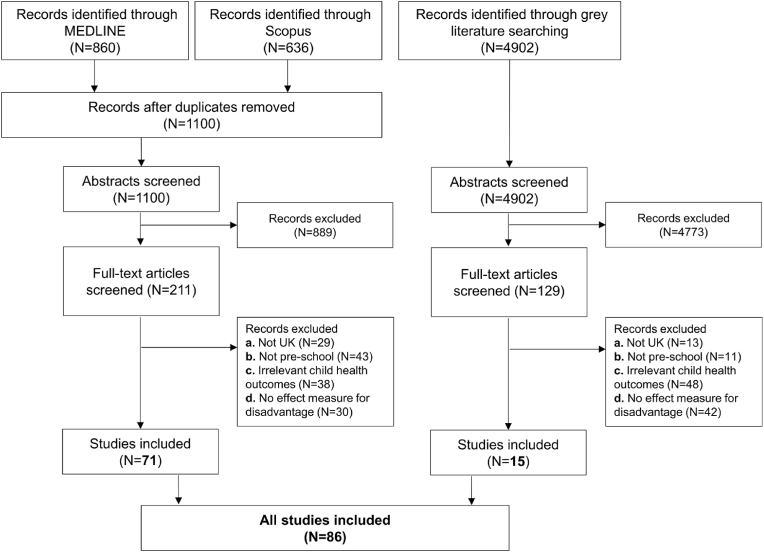
PRISMA flowchart for included studies.

We identified 6002 records for eligibility assessment, of which 340 full-text articles were screened for inclusion. A total of 86 records were included in the final analysis, 71 from peer-reviewed journals and 15 from the grey literature (Fig. 2). One study (Chiat & Polisenska, 2016) met the inclusion criteria but was subsequently excluded because the measures of disadvantage used were not sufficiently described.

### 3.2 Description of studies

Cohort data was the most common type of data analysed (34 studies; 38.6%). A quarter of the studies (22; 25.0%) specifically used Millennium Cohort Study data (Connelly & Platt, 2014). Cross sectional or administrative data were used in 26 (29.5%) and 21 (23.9%) studies respectively. One study (Centre for Excellence and Outcomes in Children and Young People's Services, 2010) used multiple types of data in their analysis, while five studies (Children's Rights Alliance for England, 2019; Early Intervention Foundation, 2017; Save the Children, 2010; The Nuffield Foundation, 2015; Welsh Assembly Government, 2011) only reviewed previous analyses, therefore a data type was not recorded. Of the four HCP high impact areas, 44 studies (48.4%) explored child development (area 6), 19 studies (20.9%) obesity (area 4), and 14 studies for both breastfeeding (area 3) and hospitalisations (area 5; Appendix II).

Of the 85 studies defining disadvantage, 34 (40.0%) measured disadvantage at the individual-level, 30 (35.3%) at area-level, and 21 (24.7%) at both. Individual-level indicators were measured at the parental/family level. Area-level indicators were measured at the neighbourhood or school-level (where reception year children were studied). The size of the area varied from a population median of 750 (Scottish data zone level) to 5000 (electoral ward level). The most common area-level indicator was the English Index of Multiple Deprivation (IMD) with a median population of 1500 per area (Ministry of Housing Communities & Local Government, 2019). Administrative data studies mostly used area-level indicators whilst cohort studies were more likely to use individual-level indicators. More than half the studies (57.6%) reported on two or more indicators of disadvantage. Across all studies, disadvantage was measured 214 times, using 17 different indicators, most commonly at the individual-level. The most frequently measured indicator was IMD (area-level; n = 42; 19.6%) followed by education (individual-level; n = 36; 16.8%) and income (individual-level; n = 34; 15.9%; Table 1).

**Table 1:** Summary of disadvantage indicators measured across all studies.

	Number of studies in which indicator was measured, n (%)
	N = 214
**Individual-level**	163 (76.2)
Education	36 (16.8)
Maternal age	3 (1.4)
Material hardship	9 (4.2)
Housing	19 (8.9)
Cohabiting status	13 (6.1)
Income	34 (15.9)
Benefits	5 (2.3)
Financial hardship	3 (1.4)
Occupation	23 (10.7)
Employment	10 (4.7)
Free school meals	8 (3.7)
**Areal-level**	**48 (22.4)**
IMD	42 (19.6)
IDACI	4 (1.9)
Child poverty index	2 (0.9)
**Other**^a^	**3 (1.4)**

a “Other” group includes indicators that were only measured in a single study, and were therefore excluded from our framework.

### 3.3 Conceptual framework for disadvantage

**Fig. 3:**
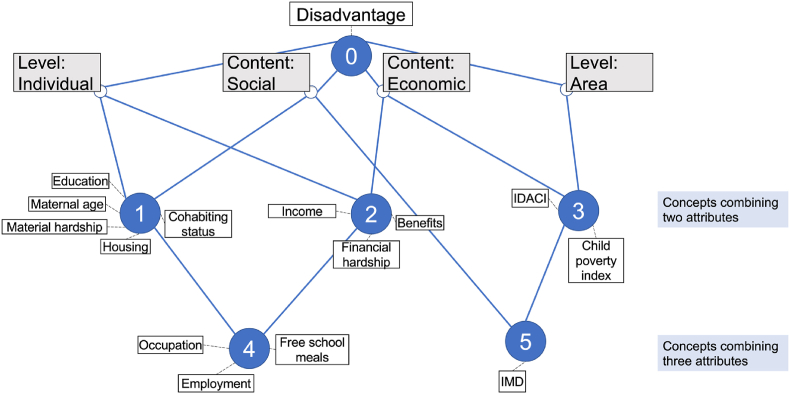
Our conceptual framework organising indicators used to measure disadvantage Each node represents a different concept: (1) individual-level social disadvantage, (2) individual-level economic disadvantage, (3) area-level economic disadvantage, (4) individual-level socioeconomic disadvantage, (5) area-level socioeconomic disadvantage. IMD = Index of Multiple Deprivation, IDACI = Income Deprivation Affecting Children Index.

Of the 17 different indicators used in studies to measure disadvantage, 14 were used by more than one study (Table 1). We organised these 14 indicators of disadvantage into a conceptual framework grouped into two broad domains, each with two attributes, described below (Fig. 3).

#### 3.3.1 Level of disadvantage indicator

The first domain, level of disadvantage indicator, includes individual-level and area-level concepts. Exploring studies that assessed both indicator types provided insight into their different conceptualisations. Most studies made a distinction between the different indicator types, for example, recognising “family” or “household” as different from “neighbourhood”, “area” or “other” disadvantage (Ajetunmobi et al., 2014; Centre for Excellence and Outcomes in Children and Young People's Services, 2010; Emerson, 2009; Emerson et al., 2009; Gore, Emerson, & Brady, 2015; Hawkins, Cole, & Law, 2009; Institute of Health Equity, 2020; Letts, Edwards, Sinka, Schaefer, & Gibbons, 2013; Marryat, Thompson, Minnis, & Wilson, 2015; Northern Ireland Office of the First Minister and Deputy First Minister, 2010; Oakley, Henderson, Redshaw, & Quigley, 2014; Paisi et al., 2018; Public Health England, 2019; Stewart, Campbell, & Gambaro, 2019; Sylva, Stein, Leach, Barnes, & Malmberg, 2011; The Sutton Trust, 2010). However, a few studies, particularly those that looked at a wide range of factors that may influence child health beyond disadvantage, did not make clear conceptual distinctions between the level used to describe disadvantage, and some grouped these multiple indicators into one (Camacho, Straatmann, Day, & Taylor-Robinson, 2019; Emerson et al., 2014; Gonzalez-Gomez, O'Brien, & Harris, 2020; McGillion, Pine, Herbert, & Matthews, 2017).

#### 3.3.2 Content of disadvantage indicator

The second domain, content of the disadvantage indicator, includes social and economic concepts. Several studies focussed specifically on economic aspects of disadvantage, using terminology such as “poverty”, measured using indicators of income or financial hardship (Save the Children, 2010; Dickerson & Popli, 2016; Holmes & Kiernan, 2013; Kelly, Sacker, Del Bono, Francesconi, & Marmot, 2011; Save the; Schoon, Jones, Cheng, & Maughan, 2012; The Nuffield Foundation, 2015; Violato, Petrou, Gray, & Redshaw, 2011). Other studies focused primarily on social aspects of disadvantage, e.g., Goncalves (2017) used the term “social disadvantage” when measuring indicators including cohabiting status and maternal age (Goncalves, 2017; J. Law, Clegg, Rush, Roulstone, & Peters, 2019; McGillion et al., 2017).

Most studies combined social and economic disadvantage, reflected in the frequently used term “socioeconomic status”. In using this term, studies fell into two categories: (1) those that measured disadvantage using the indicator “The National Statistics Socio-economic classification”, a measure of occupation published by the UK Office for National Statistics, e.g., Gray, Hernandez Alava, Kelly, and Campbell (2018), or (2) those that used the term “socioeconomic status” to summarise a range of indicators. For example, Letts et al. (2013) do not assess occupation, but use the term “socioeconomic status” to describe indicators of maternal education and IMD.

The framework illustrated in Fig. 3 explores the relationships between these domains. Attributes from each domain have been combined into nodes that reflect indicators of disadvantage that are conceptually similar. These are as follows:

Indicators combining 2 attributes:

- Node 1: individual-level and social concepts, e.g., housing
- Node 2: individual-level and economic concepts, e.g., income
- Node 3: area-level and economic concepts, e.g., Child Poverty Index

Indicators combining 3 attributes:

- Node 4: individual-level, social, and economic concepts, e.g., occupation
- Node 5: area-level, social, and economic concepts, e.g., IMD.

Studies that assessed multiple indicators of disadvantage typically selected indicators from different nodes. For example, common indicators measured together were education, income, and occupation which fall into nodes 1, 2 and 4 respectively.

### 3.4 Associations between disadvantage and child health outcomes

Overall, 80 studies presented new data on the association between disadvantage and child health outcomes. The remaining 6 studies reviewed pre-existing analyses that explored the associations between disadvantage and child health and were not included in our results here. The majority of studies (50; 62.5%) found statistically significant evidence for an association between greater disadvantage and poorer child health outcomes. Twenty studies (25.0%) found the evidence for an association varied according to which indicator of disadvantage was being assessed, and 10 studies (12.5%) found the association between disadvantage and poor child health outcomes was not statistically significant (however, the direction suggested disadvantage was associated with poorer outcomes).

**Fig. 4:**
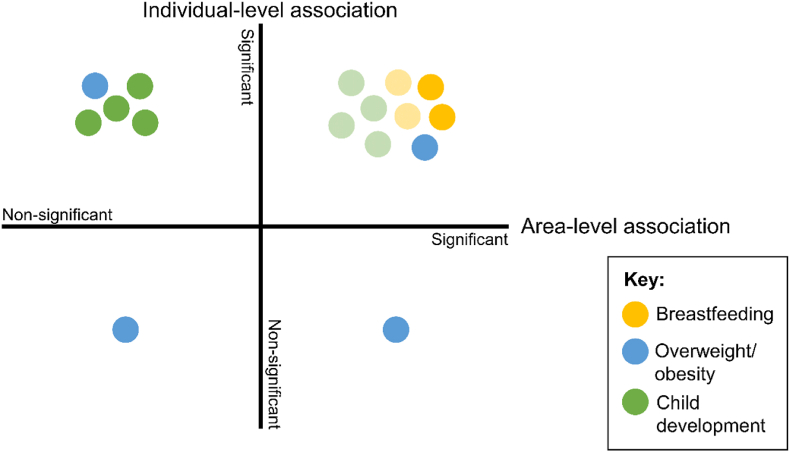
A comparison of the associations identified between disadvantage and child health outcomes when using individual-level and area-level indicators to measure disadvantage. Each circle represents one study, colour-coded according to the child health outcome that the study explored. **Upper left quadrant**: studies that reported significant associations only at the individual-level; **upper right quadrant**: studies that reported significant associations using both indicator types. The shaded circles are studies for which the area-level association reported was weaker than the individual-level; **lower left quadrant**: study that reported non-significant associations for both individual- and area-level indicators; **lower right quadrant**: study that reported significant associations only at the area-level. . (For interpretation of the references to colour in this figure legend, the reader is referred to the Web version of this article.)

To compare the associations identified using individual-level and area-level indicators of disadvantage, we looked at the studies in the review that measured both. 21 studies measured disadvantage using both individual- and area-level indicators. Five could not be used because they combined or presented indicators in such a way that we could not interpret them separately (Emerson et al., 2014; Gonzalez-Gomez et al., 2020; McGillion et al., 2017; Public Health England, 2019; Stewart et al., 2019). Therefore, we compared associations for 16 studies. These fell into four categories (Fig. 4): (1) 9 studies found a significant association between disadvantage and the child health outcome both at the area-level and individual-level, 6 of these had weaker associations using area-level indicators compared to individual-level (Ajetunmobi et al., 2014; Brown, Raynor, Benton, & Lee, 2010; Centre for Excellence and Outcomes in Children and Young People's Services, 2010; Emerson, 2009; Emerson et al., 2009; Gore et al., 2015; Institute of Health Equity, 2020; Letts et al., 2013; Oakley et al., 2014), (2) 1 study reported an association at the area-level, but not at the individual-level (Paisi et al., 2018), (3) 5 studies reported an association at the individual-level but not at the area-level (Camacho et al., 2019; Deputy First Minister, 2010; Marryat et al., 2015; Northern Ireland Office of the First Minister and; Sylva et al., 2011; The Sutton Trust, 2010), and (4) 1 study found no association at the individual- nor area-level (Hawkins et al., 2009). Studies that explored obesity had the most varied findings, with a study represented in each of the four categories. No studies used both indicator types while assessing hospitalisation as an outcome.

## 4 Discussion

Our framework conceptualising disadvantage as measured in the literature to describe and quantify health inequalities in the early years demonstrates substantial variation in the way disadvantage is measured. We highlight the multi-dimensionality and range of indicators used to measure disadvantage and identify populations at risk of poor child health outcomes. These indicators span two domains each with two attributes (social, economic, individual, and area) that shape childhood disadvantage.

Our analysis of how individual- and area-level indicators influence the associations between disadvantage and child health shows that while area-level indicators of disadvantage are commonly used, these tend to show weaker associations than individual-level indicators.

Choice of indicator is likely to have implications for research, practice, and policy. For example, using area-level indicators could make it more difficult to quantify the effect of interventions aimed at reducing inequalities. Indicators should be carefully considered based on the context when designing, delivering, and interpreting evidence-based interventions for the early years.

### 4.1 Conceptual framework

Despite widespread use of the term disadvantage, we show variation and nuance in the way it is measured in the studies reviewed. We also found that multiple indicators are commonly used to describe disadvantage within the same study. Our conceptual framework categorises disadvantage into two domains (content and level of indicator), each with two attributes (economic, social, individual and area).

Economic factors are a key aspect of disadvantage. Many of the studies we reviewed used individual-level, economic indicators (as summarised in node 2 of the framework) to describe disadvantage. This is in line with previous literature focussing on measuring income in health research (Galobardes et al., 2007). Evidence suggests that economic disadvantage has a direct causal effect on child health outcomes (Cooper & Stewart, 2021), but we also show that in the child health context other household measures such as financial hardship may also be considered beyond income alone.

Most of the studies in our review also use social indicators to measure disadvantage. These are closely aligned with the indicators of disadvantage explored in the social science literature, namely, education, housing, and neighbourhood inequalities (Platt, 2011). In the child health literature, we additional see family factors such as maternal age and cohabiting or marital status as frequently used indicators (framework node 1).

### 4.2 Association analysis

In the studies reviewed, we confirmed that disadvantage is associated with worse early years child health outcomes, regardless of disadvantage indicator used. We found the strength of associations varied according to whether an individual or area-level measure was used. Individual-level indicators may be better at characterising risk factors for poor child health outcomes, as they tend to result in stronger associations. Area-level indicators, on the other hand, may underestimate the relationship between disadvantage and child health in the early years. These findings support previous research suggesting that area-level indicators may not be good proxies for exploring associations with outcomes at the individual-level (Clelland & Hill, 2019; Pardo-Crespo et al., 2013).

Area-level measures are more readily available for use in research, particularly in population-level administrative data (Jerrim, 2021; Ministry of Housing Communities & Local Government, 2019). Almost 40% of the studies we reviewed used cohort data, for which individual-level indicators of disadvantage are available. Administrative data can offer a more representative picture of services and interventions nationally, however, typically only have area-level indicators of disadvantage available (primarily IMD). Initiatives to link individual-level indicators of disadvantage such as housing, for example through the Unique Property Reference Number, or parental education, could provide more granular data using administrative sources to enable more effective targeting and evaluation of interventions (Geospatial Commission, 2020; Mc Grath-Lone et al., 2022).

### 4.3 Choosing indicators to measure disadvantage

Choice of indicator remains a complex issue and should be carefully considered based on the research, practice, and policy context. Recent research has shown that the strength of association between disadvantage and child health varies based on both the child development outcome assessed and the indicator of disadvantage used (Cattan et al., 2022). This highlights the importance of context when choosing indicators when evaluating and implementing interventions. Whilst area-level indicators such as IMD may be a useful indicator for informing implementation of interventions targeted at particular areas, policies targeted at individuals should be informed by indicators of disadvantage captured at the individual-level. These two concepts can work in tandem, such as interventions that use a proportionate universalism approach (such as the Healthy Child Programme), which allow for universal delivery to an area, which is further targeted at the individual-level (Cowley et al., 2018).

Choice of indicator may also be restricted by the data available, particularly those that use administrative data sources, as we have shown in this review. Although in cohort studies there may be greater control and rationale for the indicators selected to be collected from participants, administrative sources are led by the data collected for policy purposes. While this has its limitations, discussed in 4.1.2, using these indicators for research purposes can also allow researchers to ensure their evidence remains relevant to policy and practice and acceptable to the public. Linking these administrative datasets to research studies (as we have seen with the MCS studies in this review, and more recent examples such as the Early Life Cohort Feasibility study) can enhance disadvantage indicators available (Goodman, Calderwood, & Fearon, 2021). Availability of data must be uniform and accessible for researchers, practitioners and service planners alike, so that the measures of disadvantage used to evidence interventions are also accurately used for implementation (Bailey, 2015; Wickham et al., 2016).

Using the framework that we develop in this review, we highlight that disadvantage should be measured across all nodes when researching and planning interventions, to identify the most relevant target population for interventions in the early years. We also recommend, as advised in previous literature (Connelly et al., 2016), that disadvantage is measured using existing and widely used indicators, and in combinations outlined in our framework, that are most relevant to their outcomes of interest.

### 4.4 Limitations of this study

Our use of scoping review methodology resulted in considerable heterogeneity between studies. The aim of this was to identify as wide a range of indicators of disadvantage as possible. As such, we did not formally assess differences in the association between disadvantage and child health based on indicator used. A deeper understanding of how choice of disadvantage indicator(s) influences the association with child health would be valuable in helping target services and help guide choice of indicators for use in different contexts. This would be of interest for future research.

This review focuses on the measures of disadvantage that are readily collected and measured in data that can be harnessed for evaluating and planning interventions. We recognise that these measures of disadvantage are crude, and a much wider range of factors influence the relationship between disadvantage and child health outcomes, from parental health to structural inequalities. Frontline staff play a key role in assessing and understanding how disadvantage plays a role in the context of each family, which cannot be fully measured in the literature.

Policy on health is a devolved issue, and while we focus on all four nations of the UK in this paper, many studies used English data only. Researchers and policymakers in each of the devolved nations should consider their local data and early years interventions that may result in differences in the definitions and indicators of disadvantage available and used in context.

## 5 Conclusion

In this review, we develop a conceptual framework to map how disadvantage in the early years is measured in the literature, for use by researchers and policymakers to help bridge theory into practice. We highlight the need to carefully consider choice of indicators that represent all concepts of disadvantage across our framework, and that in practice this can often be limited by data availability. Better access to individual-level indicators in administrative data could further support development and implementation of interventions aimed at reducing child health inequalities in the early years, and further research is needed to understand whether access to these more sensitive measures is acceptable.

## Funding

This work was supported by a Child Health Research Charitable Incorporated Organisation PhD Studentship [award number 563654] and in part by the NIHR Great Ormond Street Hospital Biomedical Research Centre. Jenny Woodman is in part supported by the 10.13039/501100000272National Institute for Health Research (NIHR) Children and Families Policy Research Unit (PR-PRU-1217-21301). The views expressed are those of the author(s) and not necessarily those of the NIHR or the Department of Health and Social Care.

## Author contributions

**AC:** conceptualisation, data curation, formal analysis, writing - original draft. **CG:** data curation, writing - reviewing & editing. **KH:** conceptualisation, supervision, writing - reviewing & editing. **HB:** conceptualisation, supervision, writing - reviewing & editing. **JW:** conceptualisation, supervision, writing - reviewing & editing.

## Declaration of competing interest

The authors declare that they have no competing interests.
