# Supplementary material for: Measuring disadvantage in the early years in the UK: A systematic scoping review
Source: SSM Popul Health. 2022 Aug 15;19:101206. doi: 10.1016/j.ssmph.2022.101206 (PMC9465426; doi:10.1016/j.ssmph.2022.101206)
Supplement: Multimedia component 1 [file mmc1.docx]

1. Summary of search strategies

**Medline (Ovid)**

|  | **Search terms** | **Results** |
| --- | --- | --- |
| **1** | exp United Kingdom/ | 368611 |
| **2** | (english not ((published or publication* or translat* or written or language* or speak* or literature or citation*) adj5 english)).ti,ab. | 96161 |
| **3** | (gb or "g.b." or britain* or (british* not "british columbia") or uk or "u.k." or united kingdom* or (england* not "new england") or "northern ireland*" or "northern irish*" or scotland* or scottish* or ((wales or "south wales") not "new south wales") or welsh*).ti,ab,jw,in. | 2113972 |
| **4** | 1 or 2 or 3 | 2357529 |
| **5** | (exp africa/ or exp americas/ or exp antarctic regions/ or exp arctic regions/ or exp asia/ or exp oceania/) not (europe/ or exp united kingdom/) | 2927481 |
| **6** | 4 not 5 | 2240102 |
| **7** | (Poverty or depriv* or socioeconomic or socio-economic or disadvantage* or "social exclusion" or "socially excluded" or inequalit* or inequit* or underprivileg* or "social* marginali*").ab,kf,ti. or social marginalization/ or exp socioeconomic factors/ or health status disparities/ or Healthcare Disparities/ | 716925 |
| **8** | ("early years" or "pre-school child*" or "preschool child*" or infant* or "under 5s" or "under fives").ab,kf,ti. or child, preschool/ or infant/ or infant, newborn/ | 1704182 |
| **9** | ("breastfeeding" or "breast feeding" or "infant feeding").ab,kf,ti. or Breast Feeding/ | 57099 |
| **10** | ("infant growth" or "child weight" or "infant nutrition" or "childhood nutrition" or "childhood obesity").ab,kf,ti. or Pediatric Obesity/ | 24588 |
| **11** | (hospitali* or "uninten* injur*" or (accident* adj2 home)).ab,kf,ti. or "Wounds and Injuries"/ or Accidents/ | 353647 |
| **12** | ("school readiness" or (school adj2 ready) or (cogniti* adj2 development) or "brain development" or "language development" or "speech and language" or (prepar* adj2 school) or "child development" or "childhood development").ab,kf,ti. or exp Child Development/ | 102044 |
| **13** | 9 or 10 or 11 or 12 | 527929 |
| **14** | 6 and 7 and 8 and 13 | 1777 |
| **15** | 14 and 2009:2021.(sa_year). | 860 |

**Scopus**

( ( ( TITLE-ABS-KEY ( ( english AND NOT ( english W/5 ( published OR publication* OR translat* OR written OR language* OR speak* OR literature OR citation* ) ) ) ) ) OR ( TITLE-ABS-KEY ( gb OR "g.b." OR britain* OR ( british* AND NOT "british columbia" ) OR ( england* AND NOT "new england" ) OR uk OR "u.k." OR "united kingdom*" ) ) OR ( TITLE-ABS-KEY ( "northern ireland*" OR "northern irish*" OR scotland* OR scottish* OR ( ( wales OR "south wales" ) AND NOT "new south wales" ) OR welsh* ) ) ) AND NOT ( TITLE-ABS-KEY ( ( africa OR americas OR "antarctic regions" OR "arctic regions" OR "asia" OR "oceania" ) AND NOT ( europe OR "united kingdom" ) ) ) ) AND ( TITLE-ABS-KEY ( poverty OR depriv* OR socioeconomic OR "socio-economic" OR disadvantage* OR "social exclusion" OR "socially excluded" OR inequalit* OR inequit* OR underprivileg* OR "social* marginali*" OR "health status disparities" OR "healthcare disparities" ) ) AND ( TITLE-ABS-KEY ( "early years" OR "pre-school child*" OR "preschool child*" OR infant* OR "under 5s" OR "under fives" OR "newborn" ) ) AND ( ( TITLE-ABS-KEY ( "infant growth" OR "child weight" OR "infant nutrition" OR "childhood nutrition" OR "childhood obesity" OR "P*ediatric Obesity" ) ) OR ( TITLE-ABS-KEY ( "breastfeeding" OR "breast feeding" OR "infant feeding" ) ) OR ( TITLE-ABS-KEY ( hospitali* OR "uninten* injur*" OR ( accident* W/3 home ) OR "Wounds and Injuries" OR "Accident*" ) ) OR ( TITLE-ABS-KEY ( "school readiness" OR ( school W/2 ready ) OR ( cogniti* W/2 development ) OR "brain development" OR "language development" OR "speech and language" OR ( prepar* W/2 school ) OR "child development" OR "childhood development" ) ) ) AND ( LIMIT-TO ( AFFILCOUNTRY , "United Kingdom" ) ) AND ( LIMIT-TO ( PUBYEAR , 2020 ) OR LIMIT-TO ( PUBYEAR , 2019 ) OR LIMIT-TO ( PUBYEAR , 2018 ) OR LIMIT-TO ( PUBYEAR , 2017 ) OR LIMIT-TO ( PUBYEAR , 2016 ) OR LIMIT-TO ( PUBYEAR , 2015 ) OR LIMIT-TO ( PUBYEAR , 2014 ) OR LIMIT-TO ( PUBYEAR , 2013 ) OR LIMIT-TO ( PUBYEAR , 2012 ) OR LIMIT-TO ( PUBYEAR , 2011 ) OR LIMIT-TO ( PUBYEAR , 2010 ) OR LIMIT-TO ( PUBYEAR , 2009 ) )

**Grey literature**

| **Website** | **Search term(s)** | **Results** |
| --- | --- | --- |
| Social Care Online | Child health | 3581 |
| Gov.uk, Public Health England | Child health | 907 |
| Children’s Commissioner | All reports | 205 |
| National Children’s Bureau | Inequality, early years, health | 100 |
| Local Government Association | Child health, early years | 75 |
| Joseph Rowntree foundation | Child poverty | 28 |
| The Nuffield Foundation | Early years | 6 |

1. Table of characteristics of included studies

| **Study** | **HCP high impact area** | | | | **Disadvantage indicator level** | **Data type** | | | | **Disadvantage measure** | | | | | | | | | | | | | | |
| --- | --- | --- | --- | --- | --- | --- | --- | --- | --- | --- | --- | --- | --- | --- | --- | --- | --- | --- | --- | --- | --- | --- | --- | --- |
|  | **3: breastfeeding** | **4: overweight/ obesity** | **5: hospitalisations** | **6: child development** |  | **Administrative** | **Cohort** | **Cross-sectional** | **Other** | **Education** | **Maternal age** | **Material hardship** | **Housing** | **Cohabiting status** | **Income** | **Benefits** | **Financial hardship** | **Occupation** | **Employment** | **Free school meals** | **IMD** | **IDACI** | **Child poverty index** | **Other** |
| Department for Education (2013) | X |  |  | X | Individual |  |  | X |  | X |  |  |  | X | X |  |  |  | X |  |  |  |  |  |
| Emmett and Jones (2014) | X |  |  |  | Individual |  | X |  |  | X |  |  | X |  |  |  | X |  |  |  |  |  |  |  |
| Goncalves (2017) | X |  |  |  | Individual |  |  | X |  | X | X |  |  | X |  |  |  | X |  |  |  |  |  |  |
| Simpson, Quigley, Kurinczuk, and Carson (2019) | X |  |  |  | Individual |  |  | X |  | X | X |  |  | X |  |  |  | X |  |  |  |  |  |  |
| (Brown & Lee, 2011) | X |  |  |  | Individual |  |  | X |  | X |  |  | X | X | X |  |  | X |  |  |  |  |  |  |
| Schrempft, van Jaarsveld, Fisher, and Wardle (2013) | X |  |  |  | Individual |  | X |  |  | X |  |  |  |  |  |  |  |  |  |  |  |  |  |  |

| **Study** | **HCP high impact area** | | | | | **Disadvantage indicator level** | | **Data type** | | | | | | | | **Disadvantage measure** | | | | | | | | | | | | | | | | | | | | | | | | | | | | |
| --- | --- | --- | --- | --- | --- | --- | --- | --- | --- | --- | --- | --- | --- | --- | --- | --- | --- | --- | --- | --- | --- | --- | --- | --- | --- | --- | --- | --- | --- | --- | --- | --- | --- | --- | --- | --- | --- | --- | --- | --- | --- | --- | --- | --- |
|  | **3: breastfeeding** | | **4: overweight/ obesity** | **5: hospitalisations** | **6: child development** |  | | **Administrative** | | **Cohort** | | **Cross-sectional** | | **Other** | | **Education** | | **Maternal age** | | **Material hardship** | | **Housing** | | **Cohabiting status** | | **Income** | | **Benefits** | | **Financial hardship** | | **Occupation** | | **Employment** | | **Free school meals** | | **IMD** | | **IDACI** | | **Child poverty index** | | **Other** |
| Skafida (2009) | X |  | |  |  | Individual |  | |  | | X | |  | | X | |  | |  | |  | |  | |  | |  | |  | | X | |  | |  | |  | |  | |  | |  | |
| D. Baker, Garrow, and Shiels (2011) | X | |  |  |  | Area | | X | |  | |  | |  | |  | |  | |  | |  | |  | |  | |  | |  | |  | |  | |  | |  | | X | |  | |  |
| Agboado, Michel, Jackson, and Verma (2010) | X | |  |  |  | Area | | X | |  | |  | |  | |  | |  | |  | |  | |  | |  | |  | |  | |  | |  | |  | | X | |  | |  | |  |
| Henderson and Redshaw (2011) | X | |  |  |  | Area | |  | |  | | X | |  | |  | |  | |  | |  | |  | |  | |  | |  | |  | |  | |  | | X | |  | |  | |  |
| Ajetunmobi et al. (2014) | X | |  |  |  | Both | | X | |  | |  | |  | |  | |  | |  | |  | |  | |  | |  | |  | | X | |  | |  | | X | |  | |  | |  |
| Brown, Raynor, Benton, and Lee (2010) | X | |  |  |  | Both | |  | |  | | X | |  | | X | |  | |  | | X | | X | | X | |  | |  | | X | |  | |  | | X | |  | |  | |  |

| **Study** | **HCP high impact area** | | | | | | | | **Disadvantage indicator level** | | **Data type** | | | | | | | | **Disadvantage measure** | | | | | | | | | | | | | | | | | | | | | | | | | | | | | |
| --- | --- | --- | --- | --- | --- | --- | --- | --- | --- | --- | --- | --- | --- | --- | --- | --- | --- | --- | --- | --- | --- | --- | --- | --- | --- | --- | --- | --- | --- | --- | --- | --- | --- | --- | --- | --- | --- | --- | --- | --- | --- | --- | --- | --- | --- | --- | --- | --- |
|  | **3: breastfeeding** | | **4: overweight/ obesity** | | **5: hospitalisations** | | **6: child development** | |  | | **Administrative** | | **Cohort** | | **Cross-sectional** | | **Other** | | **Education** | | **Maternal age** | | **Material hardship** | | **Housing** | | **Cohabiting status** | | **Income** | | **Benefits** | | **Financial hardship** | | **Occupation** | | **Employment** | | **Free school meals** | | **IMD** | | **IDACI** | | **Child poverty index** | | **Other** | |
| Oakley, Henderson, Redshaw, and Quigley (2014) | X | |  | |  | |  | | Both | |  | |  | | X | |  | | X | |  | |  | |  | |  | |  | |  | |  | |  | |  | |  | | X | |  | |  | |  | |
| Gore, Emerson, and Brady (2015) | X | |  | |  | |  | | Both | |  | | X | |  | |  | |  | |  | | X | | X | |  | | X | |  | |  | |  | | X | |  | | X | |  | |  | |  | |
| Gray, Hernandez Alava, Kelly, and Campbell (2018) |  | | X | |  | |  | | Individual | |  | | X | |  | |  | | X | |  | |  | |  | | X | |  | |  | |  | | X | |  | |  | |  | |  | |  | |  | |
| Howe et al. (2011) |  | | X | |  | |  | | Individual | |  | | X | |  | |  | | X | |  | |  | |  | |  | |  | |  | |  | |  | |  | |  | |  | |  | |  | |  | |
| Panico, Bartley, Kelly, McMunn, and Sacker (2019) | |  | | X | |  | |  | | Individual | |  | | X | |  | |  | | X | |  | | X | | X | | X | | X | |  | |  | |  | |  | |  | |  | |  | |  | |  |

| **Study** | **HCP high impact area** | | | | **Disadvantage indicator level** | **Data type** | | | | **Disadvantage measure** | | | | | | | | | | | | | | |
| --- | --- | --- | --- | --- | --- | --- | --- | --- | --- | --- | --- | --- | --- | --- | --- | --- | --- | --- | --- | --- | --- | --- | --- | --- |
|  | **3: breastfeeding** | **4: overweight/ obesity** | **5: hospitalisations** | **6: child development** |  | **Administrative** | **Cohort** | **Cross-sectional** | **Other** | **Education** | **Maternal age** | **Material hardship** | **Housing** | **Cohabiting status** | **Income** | **Benefits** | **Financial hardship** | **Occupation** | **Employment** | **Free school meals** | **IMD** | **IDACI** | **Child poverty index** | **Other** |
| Wijlaars, Johnson, van Jaarsveld, and Wardle (2011) |  | X |  |  | Individual |  |  | X |  | X |  |  |  |  |  |  |  | X |  |  |  |  |  |  |
| Black, Joseph, Mott, and Maheswaran (2018) |  | X |  |  | Area |  |  | X |  |  |  |  |  |  |  |  |  |  |  |  | X |  |  |  |
| Ells et al. (2015) |  | X |  |  | Area |  |  | X |  |  |  |  |  |  |  |  |  |  |  |  | X |  |  |  |
| National Children's Bureau (2015) |  | X | X | X | Area | X |  |  |  |  |  |  |  |  |  |  |  |  |  |  | X |  |  |  |
| Noonan (2018) |  | X |  |  | Area |  |  | X |  |  |  |  |  |  |  |  |  |  |  |  | X |  |  |  |
| Royal College of Paediatrics and Child Health (2020) |  | X |  |  | Area |  |  | X |  |  |  |  |  |  |  |  |  |  |  |  | X |  |  |  |

| **Study** | **HCP high impact area** | | | | **Disadvantage indicator level** | **Data type** | | | | | | | **Disadvantage measure** | | | | | | | | | | | | | | | | | | | | | | | | | | | | | |
| --- | --- | --- | --- | --- | --- | --- | --- | --- | --- | --- | --- | --- | --- | --- | --- | --- | --- | --- | --- | --- | --- | --- | --- | --- | --- | --- | --- | --- | --- | --- | --- | --- | --- | --- | --- | --- | --- | --- | --- | --- | --- | --- |
|  | **3: breastfeeding** | **4: overweight/ obesity** | **5: hospitalisations** | **6: child development** |  | **Administrative** | **Cohort** | | **Cross-sectional** | | **Other** | | **Education** | | **Maternal age** | | **Material hardship** | | **Housing** | | **Cohabiting status** | | **Income** | | **Benefits** | | **Financial hardship** | | **Occupation** | | **Employment** | | **Free school meals** | | **IMD** | | **IDACI** | | **Child poverty index** | | **Other** | |
| Rudolf et al. (2019) |  | X |  |  | Area |  |  | | X | |  | |  | |  | |  | |  | |  | |  | |  | |  | |  | |  | |  | | X | |  | |  | |  | |
| Townsend, Rutter, and Foster (2012) |  | X |  |  | Area |  |  | | X | |  | |  | |  | |  | |  | |  | |  | |  | |  | |  | |  | | X | |  | |  | |  | | X | |
| Viner and Hargreaves (2019) |  | X |  |  | Area |  |  | | X | |  | |  | |  | |  | |  | |  | |  | |  | |  | |  | |  | |  | | X | | X | |  | |  | |
| Paisi, Kay, Kaimi, Witton, Nelder, Christophi, et al. (2018) |  | X |  |  | Area |  |  | | X | |  | |  | |  | |  | |  | |  | |  | | X | |  | |  | |  | |  | | X | |  | |  | |  | |
| Pallan, Adab, Sitch, and Aveyard (2014) |  | X |  |  | Area |  |  | | X | |  | |  | |  | |  | |  | |  | |  | |  | |  | |  | |  | |  | | X | |  | |  | |  | |
| Children's Rights Alliance for England (2019) |  | X |  | X | Area |  |  | |  | |  | |  | |  | |  | |  | |  | |  | |  | |  | |  | |  | |  | | X | |  | |  | |  | |
| Emerson (2009) |  | X |  |  | Both |  | | X | |  | |  | |  | |  | | X | |  | |  | |  | |  | |  | |  | |  | |  | | X | |  | |  | |  |

| **Study** | **HCP high impact area** | | | | | **Disadvantage indicator level** | **Data type** | | | | **Disadvantage measure** | | | | | | | | | | | | | | |
| --- | --- | --- | --- | --- | --- | --- | --- | --- | --- | --- | --- | --- | --- | --- | --- | --- | --- | --- | --- | --- | --- | --- | --- | --- | --- |
|  | **3: breastfeeding** | | **4: overweight/ obesity** | **5: hospitalisations** | **6: child development** |  | **Administrative** | **Cohort** | **Cross-sectional** | **Other** | **Education** | **Maternal age** | **Material hardship** | **Housing** | **Cohabiting status** | **Income** | **Benefits** | **Financial hardship** | **Occupation** | **Employment** | **Free school meals** | **IMD** | **IDACI** | **Child poverty index** | **Other** |
| Northern Ireland Office of the First Minister and Deputy First Minister (2010) |  | X | |  | X | Both |  | X |  |  | X |  |  | X |  | X |  |  | X | X |  |  |  |  | X |
| Paisi, Kay, Kaimi, Witton, Nelder, Potterton, et al. (2018) |  | X | |  |  | Both |  |  | X |  | X |  |  |  |  | X | X |  |  |  |  | X |  |  |  |
| Hawkins, Cole, and Law (2009) |  | X | |  |  | Both |  | X |  |  | X |  |  |  |  | X |  |  | X |  |  |  |  | X |  |
| Department for Work and Pensions (2010) |  |  | | X |  | Individual |  |  | X |  | X |  | X | X |  | X | X |  |  | X |  |  |  |  |  |

| **Study** | **HCP high impact area** | | | | **Disadvantage indicator level** | **Data type** | | | | **Disadvantage measure** | | | | | | | | | | | | | | |
| --- | --- | --- | --- | --- | --- | --- | --- | --- | --- | --- | --- | --- | --- | --- | --- | --- | --- | --- | --- | --- | --- | --- | --- | --- |
|  | **3: breastfeeding** | **4: overweight/ obesity** | **5: hospitalisations** | **6: child development** |  | **Administrative** | **Cohort** | **Cross-sectional** | **Other** | **Education** | **Maternal age** | **Material hardship** | **Housing** | **Cohabiting status** | **Income** | **Benefits** | **Financial hardship** | **Occupation** | **Employment** | **Free school meals** | **IMD** | **IDACI** | **Child poverty index** | **Other** |
| Davidson, Roberts, Wotton, and Goldacre (2010) |  |  | X |  | Individual | X |  |  |  |  |  |  |  |  |  |  |  | X |  |  |  |  |  |  |
| R. Baker, Orton, Tata, and Kendrick (2016) |  |  | X |  | Area | X |  |  |  |  |  |  |  |  |  |  |  |  |  |  | X |  |  |  |
| R. Baker, Tata, et al. (2016) |  |  | X |  | Area | X |  |  |  |  |  |  |  |  |  |  |  |  |  |  | X |  |  |  |
| Mbeledogu, Cecil, Millett, and Saxena (2015) |  |  | X |  | Area | X |  |  |  |  |  |  |  |  |  |  |  |  |  |  | X |  |  |  |
| Mok, Jones, Myerscough, Shah, and Minns (2010) |  |  | X |  | Area |  | X |  |  |  |  |  |  |  |  |  |  |  |  |  | X |  |  |  |
| O'Donnell, Parslow, and Draper (2010) |  |  | X |  | Area |  |  |  | X |  |  |  |  |  |  |  |  |  |  |  |  |  |  | X |

| **Study** | **HCP high impact area** | | | | | **Disadvantage indicator level** | **Data type** | | | | **Disadvantage measure** | | | | | | | | | | | | | | |
| --- | --- | --- | --- | --- | --- | --- | --- | --- | --- | --- | --- | --- | --- | --- | --- | --- | --- | --- | --- | --- | --- | --- | --- | --- | --- |
|  | **3: breastfeeding** | | **4: overweight/ obesity** | **5: hospitalisations** | **6: child development** |  | **Administrative** | **Cohort** | **Cross-sectional** | **Other** | **Education** | **Maternal age** | **Material hardship** | **Housing** | **Cohabiting status** | **Income** | **Benefits** | **Financial hardship** | **Occupation** | **Employment** | **Free school meals** | **IMD** | **IDACI** | **Child poverty index** | **Other** |
| Pockett, Adlard, Carroll, and Rajoriya (2011) |  |  | | X |  | Area | X |  |  |  |  |  |  |  |  |  |  |  |  |  |  | X |  |  |  |
| Public Health England (2020) |  |  | | X |  | Area | X |  |  |  |  |  |  |  |  |  |  |  |  |  |  | X |  |  |  |
| Cheung, Smith, Thurland, Duncan, and Semple (2013) |  |  | | X |  | Area | X |  |  |  |  |  |  |  |  |  |  |  |  |  |  | X |  |  |  |
| Flanagan and Stewart (2014) |  |  | | X |  | Area | X |  |  |  |  |  |  |  |  |  |  |  |  |  |  | X |  |  |  |
| Kyle et al. (2011) |  |  | | X |  | Area | X |  |  |  |  |  |  |  |  |  |  |  |  |  |  | X |  |  |  |
| Kyle, Campbell, Powell, and Callery (2012) |  |  | | X |  | Area | X |  |  |  |  |  |  |  |  |  |  |  |  |  |  | X |  |  |  |

| **Study** | **HCP high impact area** | | | | **Disadvantage indicator level** | **Data type** | | | | **Disadvantage measure** | | | | | | | | | | | | | | |
| --- | --- | --- | --- | --- | --- | --- | --- | --- | --- | --- | --- | --- | --- | --- | --- | --- | --- | --- | --- | --- | --- | --- | --- | --- |
|  | **3: breastfeeding** | **4: overweight/ obesity** | **5: hospitalisations** | **6: child development** |  | **Administrative** | **Cohort** | **Cross-sectional** | **Other** | **Education** | **Maternal age** | **Material hardship** | **Housing** | **Cohabiting status** | **Income** | **Benefits** | **Financial hardship** | **Occupation** | **Employment** | **Free school meals** | **IMD** | **IDACI** | **Child poverty index** | **Other** |
| Beauregard, Drews-Botsch, Sales, Flanders, and Kramer (2018) |  |  |  | X | Individual |  | X |  |  | X |  |  |  |  | X |  |  | X |  |  |  |  |  |  |
| Dickerson and Popli (2016) |  |  |  | X | Individual |  | X |  |  |  |  |  |  |  | X |  |  |  |  |  |  |  |  |  |
| Emerson and Brigham (2015) |  |  |  | X | Individual |  |  | X |  |  |  |  | X |  | X |  |  |  | X |  |  |  |  |  |
| Hansen and Jones (2010) |  |  |  | X | Individual |  | X |  |  | X |  |  | X | X | X |  |  | X |  |  |  |  |  |  |
| Johnson et al. (2015) |  |  |  | X | Individual |  | X |  |  | X |  |  | X | X |  |  |  | X |  |  |  |  |  |  |
| Johnson et al. (2018) |  |  |  | X | Individual |  | X |  |  | X |  |  | X | X |  |  |  | X |  |  |  |  |  |  |
| Kelly, Sacker, Del Bono, Francesconi, and Marmot (2011) |  |  |  | X | Individual |  | X |  |  |  |  |  |  |  | X |  |  |  |  |  |  |  |  |  |

| **Study** | **HCP high impact area** | | | | | **Disadvantage indicator level** | **Data type** | | | | **Disadvantage measure** | | | | | | | | | | | | | | |
| --- | --- | --- | --- | --- | --- | --- | --- | --- | --- | --- | --- | --- | --- | --- | --- | --- | --- | --- | --- | --- | --- | --- | --- | --- | --- |
|  | **3: breastfeeding** | | **4: overweight/ obesity** | **5: hospitalisations** | **6: child development** |  | **Administrative** | **Cohort** | **Cross-sectional** | **Other** | **Education** | **Maternal age** | **Material hardship** | **Housing** | **Cohabiting status** | **Income** | **Benefits** | **Financial hardship** | **Occupation** | **Employment** | **Free school meals** | **IMD** | **IDACI** | **Child poverty index** | **Other** |
| Law, Clegg, Rush, Roulstone, and Peters (2019) |  |  | |  | X | Individual |  | X |  |  | X |  | X | X |  |  |  | X | X |  |  |  |  |  |  |
| Playford, Dibben, and Williamson (2017) |  |  | |  | X | Individual | X |  |  |  | X |  |  |  |  |  |  |  | X |  |  |  |  |  |  |
| Schoon, Jones, Cheng, and Maughan (2012) |  |  | |  | X | Individual |  | X |  |  |  |  |  |  | X | X |  |  |  |  |  |  |  |  |  |
| Zilanawala, Sacker, and Kelly (2019) |  |  | |  | X | Individual |  | X |  |  |  |  |  |  |  | X |  |  |  |  |  |  |  |  |  |
| Chittleborough, Lawlor, and Lynch (2011) |  |  | |  | X | Individual |  | X |  |  | X | X |  |  | X |  |  | X |  |  |  |  |  |  |  |
| Holmes and Kiernan (2013) |  |  | |  | X | Individual |  | X |  |  |  |  |  |  |  | X |  |  |  |  |  |  |  |  |  |

| **Study** | **HCP high impact area** | | | | | **Disadvantage indicator level** | **Data type** | | | | **Disadvantage measure** | | | | | | | | | | | | | | |
| --- | --- | --- | --- | --- | --- | --- | --- | --- | --- | --- | --- | --- | --- | --- | --- | --- | --- | --- | --- | --- | --- | --- | --- | --- | --- |
|  | **3: breastfeeding** | | **4: overweight/ obesity** | **5: hospitalisations** | **6: child development** |  | **Administrative** | **Cohort** | **Cross-sectional** | **Other** | **Education** | **Maternal age** | **Material hardship** | **Housing** | **Cohabiting status** | **Income** | **Benefits** | **Financial hardship** | **Occupation** | **Employment** | **Free school meals** | **IMD** | **IDACI** | **Child poverty index** | **Other** |
| Rougeaux, Hope, Law, and Pearce (2017) |  |  | |  | X | Individual |  | X |  |  | X |  |  |  |  | X |  |  |  |  |  |  |  |  |  |
| Stein et al. (2013) |  |  | |  | X | Individual |  | X |  |  | X |  |  |  |  | X |  |  | X |  |  |  |  |  |  |
| Violato, Petrou, Gray, and Redshaw (2011) |  |  | |  | X | Individual |  | X |  |  |  |  |  |  |  | X |  |  |  |  |  |  |  |  |  |
| Midouhas, Yogaratnam, Flouri, and Charman (2013) |  |  | |  | X | Individual |  | X |  |  |  |  |  | X |  | X | X |  |  |  |  |  |  |  |  |
| Early Intervention Foundation (2017) |  |  | |  | X | Individual |  |  |  |  |  |  |  |  |  |  |  |  |  |  | X |  |  |  |  |
| The Nuffield Foundation (2015) |  |  | |  | X | Individual |  |  |  |  |  |  |  |  |  | X |  |  |  |  |  |  |  |  |  |

| **Study** | **HCP high impact area** | | | | | **Disadvantage indicator level** | **Data type** | | | | **Disadvantage measure** | | | | | | | | | | | | | | |
| --- | --- | --- | --- | --- | --- | --- | --- | --- | --- | --- | --- | --- | --- | --- | --- | --- | --- | --- | --- | --- | --- | --- | --- | --- | --- |
|  | **3: breastfeeding** | | **4: overweight/ obesity** | **5: hospitalisations** | **6: child development** |  | **Administrative** | **Cohort** | **Cross-sectional** | **Other** | **Education** | **Maternal age** | **Material hardship** | **Housing** | **Cohabiting status** | **Income** | **Benefits** | **Financial hardship** | **Occupation** | **Employment** | **Free school meals** | **IMD** | **IDACI** | **Child poverty index** | **Other** |
| Save the Children (2010) |  |  | |  | X | Individual | X |  |  |  |  |  |  |  |  | X |  |  |  |  | X |  |  |  |  |
| Welsh Assembly Government (2011) |  |  | |  | X | Individual |  |  |  |  | X |  |  | X |  | X |  |  | X | X | X |  |  |  |  |
| Barry et al. (2015) |  |  | |  | X | Area | X |  |  |  |  |  |  |  |  |  |  |  |  |  |  | X |  |  |  |
| Basit, Hughes, Iqbal, and Cooper (2015) |  |  | |  | X | Area |  |  | X |  |  |  |  |  |  |  |  |  |  |  |  | X |  |  |  |
| Ene et al. (2019) |  |  | |  | X | Area |  |  |  | X |  |  |  |  |  |  |  |  |  |  |  | X |  |  |  |
| Flouri and Sarmadi (2016) |  |  | |  | X | Area |  | X |  |  |  |  |  | X |  |  |  |  |  |  |  |  |  |  |  |
| Woolfson, Geddes, McNicol, Booth, and Frank (2013) |  |  | |  | X | Area |  |  | X |  |  |  |  |  |  |  |  |  |  |  |  | X |  |  |  |

| **Study** | **HCP high impact area** | | | | **Disadvantage indicator level** | **Data type** | | | | **Disadvantage measure** | | | | | | | | | | | | | | |
| --- | --- | --- | --- | --- | --- | --- | --- | --- | --- | --- | --- | --- | --- | --- | --- | --- | --- | --- | --- | --- | --- | --- | --- | --- |
|  | **3: breastfeeding** | **4: overweight/ obesity** | **5: hospitalisations** | **6: child development** |  | **Administrative** | **Cohort** | **Cross-sectional** | **Other** | **Education** | **Maternal age** | **Material hardship** | **Housing** | **Cohabiting status** | **Income** | **Benefits** | **Financial hardship** | **Occupation** | **Employment** | **Free school meals** | **IMD** | **IDACI** | **Child poverty index** | **Other** |
| Camacho, Straatmann, Day, and Taylor-Robinson (2019) |  |  |  | X | Both |  | X |  |  | X |  |  |  |  | X |  |  | X | X |  | X |  |  |  |
| Centre for Excellence and Outcomes in Children and Young People's Services (2010) |  |  |  | X | Both | X | X | X |  | X |  |  |  |  | X |  |  |  |  | X |  | X |  |  |
| Emerson et al. (2014) |  |  |  | X | Both |  | X |  |  |  |  | X | X |  | X |  |  |  | X |  | X |  |  |  |
| Emerson et al. (2009) |  |  |  | X | Both |  | X |  |  | X |  | X | X |  | X | X |  | X | X |  | X |  |  |  |
| Institute of Health Equity (2020) |  |  |  | X | Both | X |  |  |  |  |  |  |  |  |  |  |  |  |  | X | X |  |  |  |
| Letts, Edwards, Sinka, Schaefer, and Gibbons (2013) |  |  |  | X | Both |  |  | X |  | X |  |  |  |  |  |  |  |  |  |  | X |  |  |  |

| **Study** | **HCP high impact area** | | | | **Disadvantage indicator level** | **Data type** | | | | **Disadvantage measure** | | | | | | | | | | | | | | |
| --- | --- | --- | --- | --- | --- | --- | --- | --- | --- | --- | --- | --- | --- | --- | --- | --- | --- | --- | --- | --- | --- | --- | --- | --- |
|  | **3: breastfeeding** | **4: overweight/ obesity** | **5: hospitalisations** | **6: child development** |  | **Administrative** | **Cohort** | **Cross-sectional** | **Other** | **Education** | **Maternal age** | **Material hardship** | **Housing** | **Cohabiting status** | **Income** | **Benefits** | **Financial hardship** | **Occupation** | **Employment** | **Free school meals** | **IMD** | **IDACI** | **Child poverty index** | **Other** |
| Public Health England (2019) |  |  |  | X | Both | X |  |  |  |  |  |  |  |  | X |  |  |  |  | X | X |  |  |  |
| Stewart, Campbell, and Gambaro (2019) |  |  |  | X | Both | X |  |  |  |  |  |  |  |  |  |  |  |  |  | X |  | X |  |  |
| The Sutton Trust (2010) |  |  |  | X | Both |  | X |  |  | X |  | X | X |  | X |  |  |  | X |  | X |  |  |  |
| Gonzalez-Gomez, O'Brien, and Harris (2020) |  |  |  | X | Both |  | X |  |  | X |  |  |  |  | X |  |  |  |  |  | X |  |  |  |
| Marryat, Thompson, Minnis, and Wilson (2015) |  |  |  | X | Both |  | X |  |  | X |  |  |  | X | X |  |  | X |  |  | X |  |  |  |
| McGillion, Pine, Herbert, and Matthews (2017) |  |  |  | X | Both |  |  |  | X | X |  |  |  |  | X |  |  |  |  |  | X |  |  |  |
| Sylva, Stein, Leach, Barnes, and Malmberg (2011) |  |  |  | X | Both |  | X |  |  | X |  | X | X |  | X |  |  | X |  |  |  |  | X |  |
| Chiat and Polisenska (2016) |  |  |  | X | Not stated |  |  | X |  |  |  |  |  |  |  |  |  |  |  |  |  |  |  |  |

1. References

Agboado, G., Michel, E., Jackson, E., & Verma, A. (2010). Factors associated with breastfeeding cessation in nursing mothers in a peer support programme in Eastern Lancashire. *BMC Pediatrics, 10*, 3. doi:<https://doi.org/10.1186/1471-2431-10-3>

Ajetunmobi, O., Whyte, B., Chalmers, J., Fleming, M., Stockton, D., & Wood, R. (2014). Informing the 'early years' agenda in Scotland: understanding infant feeding patterns using linked datasets. *Journal of Epidemiology & Community Health, 68*(1), 83-92. doi:<https://doi.org/10.1136/jech-2013-202718>

Baker, D., Garrow, A., & Shiels, C. (2011). Inequalities in immunisation and breast feeding in an ethnically diverse urban area: cross-sectional study in Manchester, UK. *J Epidemiol Community Health, 65*(4), 346-352. doi:<https://doi.org/10.1136/jech.2008.085142>

Baker, R., Orton, E., Tata, L. J., & Kendrick, D. (2016). Epidemiology of poisonings, fractures and burns among 0-24 year olds in England using linked health and mortality data. *European Journal of Public Health, 26*(6), 940-946. doi:<https://doi.org/10.1093/eurpub/ckw064>

Baker, R., Tata, L. J., Kendrick, D., Burch, T., Kennedy, M., & Orton, E. (2016). Differing patterns in thermal injury incidence and hospitalisations among 0-4 year old children from England. *Burns, 42*(7), 1609-1616. doi:<https://doi.org/10.1016/j.burns.2016.05.007>

Barry, S. J., Marryat, L., Thompson, L., Ellaway, A., White, J., McClung, M., & Wilson, P. (2015). Mapping area variability in social and behavioural difficulties among Glasgow pre-schoolers: linkage of a survey of pre-school staff with routine monitoring data. *Child Care Health Dev, 41*(6), 853-864. doi:<https://doi.org/10.1111/cch.12237>

Basit, T. N., Hughes, A., Iqbal, Z., & Cooper, J. (2015). The influence of socio-economic status and ethnicity on speech and language development. *International Journal of Early Years Education, 23*(1), 115-133. doi:<https://doi.org/10.1080/09669760.2014.973838>

Beauregard, J. L., Drews-Botsch, C., Sales, J. M., Flanders, W. D., & Kramer, M. R. (2018). Preterm Birth, Poverty, and Cognitive Development. *Pediatrics, 141*(1), e20170509. doi:<https://doi.org/10.1542/peds.2017-0509>

Black, M., Joseph, V., Mott, L., & Maheswaran, R. (2018). Increasing inequality in childhood obesity in primary schools in a northern English town. *Public Health, 158*, 9-14. doi:<https://doi.org/10.1016/j.puhe.2018.01.033>

Brown, A., & Lee, M. (2011). A descriptive study investigating the use and nature of baby-led weaning in a UK sample of mothers. *Maternal & Child Nutrition, 7*(1), 34-47. doi:<https://doi.org/10.1111/j.1740-8709.2010.00243.x>

Brown, A., Raynor, P., Benton, D., & Lee, M. D. (2010). Indices of Multiple Deprivation predict breastfeeding duration in England and Wales. *European Journal of Public Health, 20*(2), 231-235. doi:<https://doi.org/10.1093/eurpub/ckp114>

Camacho, C., Straatmann, V. S., Day, J. C., & Taylor-Robinson, D. (2019). Development of a predictive risk model for school readiness at age 3 years using the UK Millennium Cohort Study. *BMJ Open, 9*(6), e024851. doi:<https://doi.org/10.1136/bmjopen-2018-024851>

Centre for Excellence and Outcomes in Children and Young People's Services. (2010). *Narrowing the gap in outcomes for young children through effective practices in the early years*. Retrieved from <https://www.basw.co.uk/system/files/resources/basw_123326-4_0.pdf>

Cheung, C. R., Smith, H., Thurland, K., Duncan, H., & Semple, M. G. (2013). Population variation in admission rates and duration of inpatient stay for bronchiolitis in England. *Archives of Disease in Childhood, 98*(1), 57-59. doi:<http://doi.org/10.1136/archdischild-2012-302277>

Chiat, S., & Polisenska, K. (2016). A Framework for Crosslinguistic Nonword Repetition Tests: Effects of Bilingualism and Socioeconomic Status on Children's Performance. *Journal of Speech Language & Hearing Research, 59*(5), 1179-1189. doi:<https://doi.org/10.1044/2016_JSLHR-L-15-0293>

Children's Rights Alliance for England. (2019). *State of Children’s Rights in England 2018: Health*. Retrieved from <http://www.crae.org.uk/publications-resources/state-of-childrens-rights-2018/>

Chittleborough, C. R., Lawlor, D. A., & Lynch, J. W. (2011). Young maternal age and poor child development: predictive validity from a birth cohort. *Pediatrics, 127*(6), e1436-1444. doi:<https://doi.org/10.1542/peds.2010-3222>

Davidson, R., Roberts, S. E., Wotton, C. J., & Goldacre, M. J. (2010). Influence of maternal and perinatal factors on subsequent hospitalisation for asthma in children: evidence from the Oxford record linkage study. *BMC Pulmonary Medicine, 10*, 14. doi:<https://doi.org/10.1186/1471-2466-10-14>

Department for Education. (2013). *Evaluation of children's centres in England: strand 2 - baseline survey of families using children's centres in the most disadvantaged areas*. Retrieved from <https://www.gov.uk/government/publications/evaluation-of-childrens-centres-in-england-strand-2-baseline-survey-of-families-using-childrens-centres-in-the-most-disadvantaged-areas>

Department for Work and Pensions. (2010). *Families with children in Britain: Findings from the 2008 Families and Children Study (FACS) (RR656)*. Retrieved from <https://www.gov.uk/government/publications/families-with-children-in-britain-findings-from-the-2008-families-and-children-study-facs-rr656>

Dickerson, A., & Popli, G. K. (2016). Persistent poverty and children's cognitive development: Evidence from the UK Millennium Cohort Study. *J. R. Stat. Soc. A, 179*(2), 535-558. doi:<https://doi.org/10.1111/rssa.12128>

Early Intervention Foundation. (2017). *Interparental conflict and outcomes for children in the contexts of poverty and economic pressure*. Retrieved from <https://www.eif.org.uk/report/interparental-conflict-and-outcomes-for-children-in-the-contexts-of-poverty-and-economic-pressure>

Ells, L. J., Hancock, C., Copley, V. R., Mead, E., Dinsdale, H., Kinra, S., . . . Rutter, H. (2015). Prevalence of severe childhood obesity in England: 2006-2013. *Archives of Disease in Childhood, 100*(7), 631-636. doi:<https://doi.org/10.1136/archdischild-2014-307036>

Emerson, E. (2009). Overweight and obesity in 3- and 5-year-old children with and without developmental delay. *Public Health, 123*(2), 130-133. doi:<https://doi.org/10.1016/j.puhe.2008.10.020>

Emerson, E., Blacher, J., Einfeld, S., Hatton, C., Robertson, J., & Stancliffe, R. J. (2014). Environmental risk factors associated with the persistence of conduct difficulties in children with intellectual disabilities and autistic spectrum disorders. *Research in Developmental Disabilities, 35*(12), 3508-3517. doi:<https://doi.org/10.1016/j.ridd.2014.08.039>

Emerson, E., & Brigham, P. (2015). Exposure of children with developmental delay to social determinants of poor health: cross-sectional case record review study. *Child: Care, Health & Development, 41*(2), 249-257. doi:<https://doi.org/10.1111/cch.12144>

Emerson, E., Graham, H., McCulloch, A., Blacher, J., Hatton, C., & Llewellyn, G. (2009). The social context of parenting 3-year-old children with developmental delay in the UK. *Child: Care, Health & Development, 35*(1), 63-70. doi:<https://doi.org/10.1111/j.1365-2214.2008.00909.x>

Emmett, P. M., & Jones, L. R. (2014). Diet and growth in infancy: relationship to socioeconomic background and to health and development in the Avon Longitudinal Study of Parents and Children. *Nutrition Reviews, 72*(8), 483-506. doi:<https://doi.org/10.1111/nure.12122>

Ene, D., Der, G., Fletcher-Watson, S., O'Carroll, S., MacKenzie, G., Higgins, M., & Boardman, J. P. (2019). Associations of Socioeconomic Deprivation and Preterm Birth With Speech, Language, and Communication Concerns Among Children Aged 27 to 30 Months. *JAMA network open, 2*(9), e1911027. doi:<https://doi.org/10.1001/jamanetworkopen.2019.11027>

Flanagan, C. F., & Stewart, M. (2014). Factors associated with early neonatal attendance to a paediatric emergency department. *Archives of Disease in Childhood, 99*(3), 239-243. doi:<https://doi.org/10.1136/archdischild-2013-304298>

Flouri, E., & Sarmadi, Z. (2016). Prosocial behavior and childhood trajectories of internalizing and externalizing problems: The role of neighborhood and school contexts. *Developmental Psychology, 52*(2), 253-258. doi:<https://doi.org/10.1037/dev0000076>

Goncalves, A. V. (2017). What influences women to bottle-feed from birth and to discontinue breastfeeding early? *British Journal of Midwifery, 25*(7), 442-450. doi:<https://doi.org/10.12968/bjom.2017.25.7.442>

Gonzalez-Gomez, N., O'Brien, F., & Harris, M. (2020). The effects of prematurity and socioeconomic deprivation on early speech perception: A story of two different delays. *Developmental Science*, e13020. doi:<https://doi.org/10.1111/desc.13020>

Gore, N., Emerson, E., & Brady, S. (2015). Rates of breastfeeding and exposure to socio-economic adversity amongst children with intellectual disability. *Research in Developmental Disabilities, 39*, 12-19. doi:<https://doi.org/10.1016/j.ridd.2014.12.028>

Gray, L. A., Hernandez Alava, M., Kelly, M. P., & Campbell, M. J. (2018). Family lifestyle dynamics and childhood obesity: evidence from the millennium cohort study. *BMC Public Health, 18*(1), 500. doi:<https://doi.org/10.1186/s12889-018-5398-5>

Hansen, K., & Jones, E. M. (2010). Age 5 cognitive development in England. *Child Indicators Research, 3*(1), 105-126. doi:<https://doi.org/10.1007/s12187-009-9055-5>

Hawkins, S. S., Cole, T. J., & Law, C. (2009). An ecological systems approach to examining risk factors for early childhood overweight: findings from the UK Millennium Cohort Study. *Journal of Epidemiology & Community Health, 63*(2), 147-155. doi:<https://doi.org/10.1136/jech.2008.077917>

Henderson, J., & Redshaw, M. (2011). Midwifery factors associated with successful breastfeeding. *Child: Care, Health & Development, 37*(5), 744-753. doi:<https://doi.org/10.1111/j.1365-2214.2010.01177.x>

Holmes, J., & Kiernan, K. (2013). Persistent poverty and children's development in the early years of childhood. *Policy and politics, 41*(1), 19-42. doi:<https://doi.org/10.1332/030557312X645810>

Howe, L. D., Tilling, K., Galobardes, B., Smith, G. D., Ness, A. R., & Lawlor, D. A. (2011). Socioeconomic disparities in trajectories of adiposity across childhood. *International Journal of Pediatric Obesity, 6*(2-2), e144-153. doi:<https://doi.org/10.3109/17477166.2010.500387>

Institute of Health Equity. (2020). *Health Equity in England: The Marmot Review 10 Years On*. Retrieved from <https://www.health.org.uk/publications/reports/the-marmot-review-10-years-on>

Johnson, S., Matthews, R., Draper, E. S., Field, D. J., Manktelow, B. N., Marlow, N., . . . Boyle, E. M. (2015). Early Emergence of Delayed Social Competence in Infants Born Late and Moderately Preterm. *Journal of Developmental & Behavioral Pediatrics, 36*(9), 690-699. doi:<https://doi.org/10.1097/DBP.0000000000000222>

Johnson, S., Waheed, G., Manktelow, B. N., Field, D. J., Marlow, N., Draper, E. S., & Boyle, E. M. (2018). Differentiating the Preterm Phenotype: Distinct Profiles of Cognitive and Behavioral Development Following Late and Moderately Preterm Birth. *Journal of Pediatrics, 193*, 85-92.e81. doi:<https://doi.org/10.1016/j.jpeds.2017.10.002>

Kelly, Y., Sacker, A., Del Bono, E., Francesconi, M., & Marmot, M. (2011). What role for the home learning environment and parenting in reducing the socioeconomic gradient in child development? Findings from the Millennium Cohort Study. *Archives of Disease in Childhood, 96*(9), 832-837. doi:<https://doi.org/10.1136/adc.2010.195917>

Kyle, R. G., Campbell, M., Powell, P., & Callery, P. (2012). Relationships between deprivation and duration of children's emergency admissions for breathing difficulty, feverish illness and diarrhoea in North West England: an analysis of hospital episode statistics. *BMC Pediatrics, 12*, 22. doi:<https://doi.org/10.1186/1471-2431-12-22>

Kyle, R. G., Kukanova, M., Campbell, M., Wolfe, I., Powell, P., & Callery, P. (2011). Childhood disadvantage and emergency admission rates for common presentations in London: An exploratory analysis. *Archives of Disease in Childhood, 96*(3), 221-226. doi:<http://doi.org/10.1136/adc.2009.180125>

Law, J., Clegg, J., Rush, R., Roulstone, S., & Peters, T. J. (2019). Association of proximal elements of social disadvantage with children's language development at 2 years: an analysis of data from the Children in Focus (CiF) sample from the ALSPAC birth cohort. *International Journal of Language & Communication Disorders, 54*(3), 362-376. doi:<https://doi.org/10.1111/1460-6984.12442>

Letts, C., Edwards, S., Sinka, I., Schaefer, B., & Gibbons, W. (2013). Socio-economic status and language acquisition: children's performance on the new Reynell Developmental Language Scales. *International Journal of Language & Communication Disorders, 48*(2), 131-143. doi:<https://doi.org/10.1111/1460-6984.12004>

Marryat, L., Thompson, L., Minnis, H., & Wilson, P. (2015). Exploring the social, emotional and behavioural development of preschool children: is Glasgow different? *International Journal for Equity in Health, 14*, 3. doi:<https://doi.org/10.1186/s12939-014-0129-8>

Mbeledogu, C. N. A., Cecil, E. V., Millett, C., & Saxena, S. (2015). Hospital admissions for unintentional poisoning in preschool children in England; 2000-2011. *Archives of Disease in Childhood, 100*(2), 180-182. doi:<http://doi.org/10.1136/archdischild-2013-305298>

McGillion, M., Pine, J. M., Herbert, J. S., & Matthews, D. (2017). A randomised controlled trial to test the effect of promoting caregiver contingent talk on language development in infants from diverse socioeconomic status backgrounds. *Journal of Child Psychology & Psychiatry & Allied Disciplines, 58*(10), 1122-1131. doi:<https://doi.org/10.1111/jcpp.12725>

Midouhas, E., Yogaratnam, A., Flouri, E., & Charman, T. (2013). Psychopathology trajectories of children with autism spectrum disorder: The role of family poverty and parenting. *Journal of the American Academy of Child and Adolescent Psychiatry, 52*(10), 1057-1065.e1051. doi:<https://doi.org/10.1016/j.jaac.2013.07.011>

Mok, J. Y. Q., Jones, P. A., Myerscough, E., Shah, A. R. F., & Minns, R. A. (2010). Non-accidental head injury: A consequence of deprivation? *Journal of Epidemiology and Community Health, 64*(12), 1049-1055. doi:<http://doi.org/10.1136/jech.2009.090217>

National Children's Bureau. (2015). *Poor Beginnings: Health Inequalities Among Young Children Across England*. Retrieved from <https://www.ncb.org.uk/resources-publications/poor-beginnings-health-inequalities-among-young-children-across-england>

Noonan, R. J. (2018). Prevalence of Childhood Overweight and Obesity in Liverpool between 2006 and 2012: Evidence of Widening Socioeconomic Inequalities. *International Journal of Environmental Research & Public Health, 15*(12), 22. doi:<https://doi.org/10.3390/ijerph15122612>

Northern Ireland Office of the First Minister and Deputy First Minister. (2010). *The consequences at age 7 of early childhood disadvantage in Northern Ireland and Great Britain*. Retrieved from <https://childhub.org/en/child-protection-online-library/consequences-age-7-early-childhood-disadvantage-northern-ireland>

O'Donnell, D. R., Parslow, R. C., & Draper, E. S. (2010). Deprivation, ethnicity and prematurity in infant respiratory failure in PICU in the UK. *Acta Paediatrica, International Journal of Paediatrics, 99*(8), 1186-1191. doi:<https://doi.org/10.1111/j.1651-2227.2010.01803.x>

Oakley, L. L., Henderson, J., Redshaw, M., & Quigley, M. A. (2014). The role of support and other factors in early breastfeeding cessation: an analysis of data from a maternity survey in England. *BMC Pregnancy & Childbirth, 14*, 88. doi:<https://doi.org/10.1186/1471-2393-14-88>

Paisi, M., Kay, E., Kaimi, I., Witton, R., Nelder, R., Christophi, C., & Lapthorne, D. (2018). Obesity and Dental Caries in Young Children in Plymouth, United Kingdom: A Spatial Analysis. *Community dental health, 35*(1), 58-64. doi:<https://doi.org/10.1922/CDH_4214Paisi07>

Paisi, M., Kay, E., Kaimi, I., Witton, R., Nelder, R., Potterton, R., & Lapthorne, D. (2018). Obesity and caries in four-to-six year old English children: a cross-sectional study. *BMC Public Health, 18*(1), 267. doi:<https://doi.org/10.1186/s12889-018-5156-8>

Pallan, M. J., Adab, P., Sitch, A. J., & Aveyard, P. (2014). Are school physical activity characteristics associated with weight status in primary school children? A multilevel cross-sectional analysis of routine surveillance data. *Archives of Disease in Childhood, 99*(2), 135-141. doi:<https://doi.org/10.1136/archdischild-2013-303987>

Panico, L., Bartley, M., Kelly, Y. J., McMunn, A., & Sacker, A. (2019). Family structure trajectories and early child health in the UK: Pathways to health. *Social Science and Medicine, 232*, 220-229. doi:<https://doi.org/10.1016/j.socscimed.2019.05.006>

Playford, C. J., Dibben, C., & Williamson, L. (2017). Socioeconomic disadvantage, fetal environment and child development: linked Scottish administrative records based study. *International Journal for Equity in Health, 16*(1), 203. doi:<https://doi.org/10.1186/s12939-017-0698-4>

Pockett, R. D., Adlard, N., Carroll, S., & Rajoriya, F. (2011). Paediatric hospital admissions for rotavirus gastroenteritis and infectious gastroenteritis of all causes in England: an analysis of correlation with deprivation. *Current Medical Research & Opinion, 27*(4), 777-784. doi:<https://doi.org/10.1185/03007995.2011.555757>

Public Health England. (2019). *Improving school readiness: initiatives across the south-east*. Retrieved from <https://www.gov.uk/government/publications/improving-school-readiness-initiatives-across-the-south-east>

Public Health England. (2020). *Vulnerability in childhood: a public health informed approach*. Retrieved from <https://www.gov.uk/government/publications/vulnerability-in-childhood-a-public-health-informed-approach>

Rougeaux, E., Hope, S., Law, C., & Pearce, A. (2017). Have health inequalities changed during childhood in the New Labour generation? Findings from the UK Millennium Cohort Study. *BMJ Open, 7*(1). doi:<http://doi.org/10.1136/bmjopen-2016-012868>

Royal College of Paediatrics and Child Health. (2020). *State of Child Health in the UK*. Retrieved from <https://stateofchildhealth.rcpch.ac.uk/>

Rudolf, M., Perera, R., Swanston, D., Burberry, J., Roberts, K., & Jebb, S. (2019). Observational analysis of disparities in obesity in children in the UK: Has Leeds bucked the trend? *Pediatric Obesity, 14*(9), e12529. doi:<https://doi.org/10.1111/ijpo.12529>

Save the Children. (2010). *No Child Left Behind: A Child Poverty Strategy 2011-2014*. Retrieved from <https://resourcecentre.savethechildren.net/library/no-child-left-behind-child-poverty-strategy-2011-2014>

Schoon, I., Jones, E., Cheng, H., & Maughan, B. (2012). Family hardship, family instability, and cognitive development. *Journal of Epidemiology & Community Health, 66*(8), 716-722. doi:<https://doi.org/10.1136/jech.2010.121228>

Schrempft, S., van Jaarsveld, C. H., Fisher, A., & Wardle, J. (2013). Family and infant characteristics associated with timing of core and non-core food introduction in early childhood. *European Journal of Clinical Nutrition, 67*(6), 652-657. doi:<https://doi.org/10.1038/ejcn.2013.63>

Simpson, D. A., Quigley, M. A., Kurinczuk, J. J., & Carson, C. (2019). Twenty-five-year trends in breastfeeding initiation: The effects of sociodemographic changes in Great Britain, 1985-2010. *PLoS ONE, 14*(1), e0210838. doi:<https://doi.org/10.1371/journal.pone.0210838>

Skafida, V. (2009). The relative importance of social class and maternal education for breast-feeding initiation. *Public Health Nutrition, 12*(12), 2285-2292. doi:<https://doi.org/10.1017/S1368980009004947>

Stein, A., Malmberg, L. E., Leach, P., Barnes, J., Sylva, K., & Team, F. (2013). The influence of different forms of early childcare on children's emotional and behavioural development at school entry. *Child: Care, Health & Development, 39*(5), 676-687. doi:<https://doi.org/10.1111/j.1365-2214.2012.01421.x>

Stewart, K., Campbell, T., & Gambaro, L. (2019). The peer composition of pre-school settings in England and early recorded attainment among low-income children. *British Journal of Sociology of Education, 40*(6), 717-741. doi:<https://doi.org/10.1080/01425692.2019.1583549>

Sylva, K., Stein, A., Leach, P., Barnes, J., & Malmberg, L. E. (2011). Effects of early child-care on cognition, language, and task-related behaviours at 18 months: An English study. *British Journal of Developmental Psychology, 29*(1), 18-45. doi:<https://doi.org/10.1348/026151010X533229>

The Nuffield Foundation. (2015). *Early years education and childcare: Lessons from evidence and future priorities*. Retrieved from <https://www.nuffieldfoundation.org/publications/early-years-education-and-childcare-lessons-from-evidence-and-future-priorities>

The Sutton Trust. (2010). *Low income and early cognitive development in the U.K.* Retrieved from <https://www.suttontrust.com/our-research/low-income-early-cognitive-development-u-k/>

Townsend, N., Rutter, H., & Foster, C. (2012). Age differences in the association of childhood obesity with area-level and school-level deprivation: cross-classified multilevel analysis of cross-sectional data. *International Journal of Obesity, 36*(1), 45-52. doi:<https://doi.org/10.1038/ijo.2011.191>

Viner, R. M., & Hargreaves, D. S. (2019). Trajectories of change in childhood obesity prevalence across local authorities 2007/08-2015/16: a latent trajectory analysis. *Journal of Public Health, 41*(4), 724-731. doi:<https://doi.org/10.1093/pubmed/fdy205>

Violato, M., Petrou, S., Gray, R., & Redshaw, M. (2011). Family income and child cognitive and behavioural development in the United Kingdom: does money matter? *Health Economics, 20*(10), 1201-1225. doi:<https://doi.org/10.1002/hec.1665>

Welsh Assembly Government. (2011). *Child Poverty Strategy for Wales*. Retrieved from <https://www.aber.ac.uk/en/media/departmental/sell/pdf/wellbeinghealth/Child-Poverty-Strategy-for-Wales-Feb-2010.pdf>

Wijlaars, L. P., Johnson, L., van Jaarsveld, C. H., & Wardle, J. (2011). Socioeconomic status and weight gain in early infancy. *International Journal of Obesity, 35*(7), 963-970. doi:<https://doi.org/10.1038/ijo.2011.88>

Woolfson, L. M., Geddes, R., McNicol, S., Booth, J. N., & Frank, J. (2013). A cross-sectional pilot study of the Scottish early development instrument: a tool for addressing inequality. *BMC Public Health, 13*, 1187. doi:<https://doi.org/10.1186/1471-2458-13-1187>

Zilanawala, A., Sacker, A., & Kelly, Y. (2019). Internalising and externalising behaviour profiles across childhood: The consequences of changes in the family environment. *Social Science & Medicine, 226*, 207-216. doi:<https://doi.org/10.1016/j.socscimed.2019.02.048>
